# The Epilepsy-Related Protein PCDH19 Regulates Tonic Inhibition, GABA_A_R Kinetics, and the Intrinsic Excitability of Hippocampal Neurons

**DOI:** 10.1007/s12035-020-02099-7

**Published:** 2020-09-03

**Authors:** Giulia M. Serratto, Erika Pizzi, Luca Murru, Sara Mazzoleni, Silvia Pelucchi, Elena Marcello, Michele Mazzanti, Maria Passafaro, Silvia Bassani

**Affiliations:** 1 Institute of Neuroscience, CNR, 20129 Milan, Italy; 2 Department of Bioscience, University of Milan, 20133 Milan, Italy; 3 NeuroMI Milan Center for Neuroscience, University of Milano-Bicocca, 20126 Milan, Italy; 4 Department of Medical Biotechnology and Translational Medicine, University of Milan, 20129 Milan, Italy; 5 Department of Pharmacological and Biomolecular Sciences, University of Milan, 20133 Milan, Italy

**Keywords:** PCDH19, GABA_A_R, Hyperexcitability, Intellectual disability, ASD

## Abstract

*PCDH19* encodes for protocadherin-19 (PCDH19), a cell-adhesion molecule of the cadherin superfamily preferentially expressed in the brain. *PCDH19* mutations cause a neurodevelopmental syndrome named epileptic encephalopathy, early infantile, 9 (EIEE9) characterized by seizures associated with cognitive and behavioral deficits. We recently reported that PCDH19 binds the alpha subunits of GABA_A_ receptors (GABA_A_Rs), modulating their surface availability and miniature inhibitory postsynaptic currents (mIPSCs). Here, we investigated whether PCDH19 regulatory function on GABA_A_Rs extends to the extrasynaptic receptor pool that mediates tonic current. In fact, the latter shapes neuronal excitability and network properties at the base of information processing. By combining patch-clamp recordings in whole-cell and cell-attached configurations, we provided a functional characterization of primary hippocampal neurons from embryonic rats of either sex expressing a specific PCDH19 short hairpin (sh)RNA. We first demonstrated that PCDH19 downregulation reduces GABA_A_R-mediated tonic current, evaluated by current shift and baseline noise analysis. Next, by single-channel recordings, we showed that PCDH19 regulates GABA_A_Rs kinetics without altering their conductance. In particular, GABA_A_Rs of shRNA-expressing neurons preferentially exhibit brief openings at the expense of long ones, thus displaying a flickering behavior. Finally, we showed that PCDH19 downregulation reduces the rheobase and increases the frequency of action potential firing, thus indicating neuronal hyperexcitability. These findings establish PCDH19 as a critical determinant of GABA_A_R-mediated tonic transmission and GABA_A_Rs gating, and provide the first mechanistic insights into PCDH19-related hyperexcitability and comorbidities.

**Electronic supplementary material:**

The online version of this article (10.1007/s12035-020-02099-7) contains supplementary material, which is available to authorized users.

## Introduction

Mutations in the human gene *PCDH19* result in an epileptic syndrome known as EIEE9 (epileptic encephalopathy, early infantile, 9; OMIM **#** 300088), characterized by early-onset seizures, cognitive impairment, and autistic features, in addition to a variety of behavioral problems and sleep dysregulation [1–3]. *PCDH19* is preferentially expressed in the brain, especially the limbic system and cortex [4–6], and encodes for protocadherin-19 (PCDH19), a cell-adhesion molecule of the cadherin superfamily [7, 8]. PCDH19 is supposed to mediate cell-cell recognition within neuronal circuits and their correct assembly, in accordance with PCDH19 adhesive properties [9] and recent findings that supported PCDH19 involvement in neuronal migration and sorting [10–12]. Despite these advances, most aspects of PCDH19 biological functions remain elusive, and thus the link between PCDH19 neuronal role and EIEE9 pathological phenotype.

We previously reported that PCDH19 binds the alpha subunits of GABA_A_ receptors (GABA_A_Rs) [10], ligand-gated ion channels that mediate fast inhibitory transmission in the brain [13]. GABA_A_Rs are divided between synaptic receptors that mediate phasic inhibition in response to presynaptically released GABA and extrasynaptic receptors that provide tonic inhibition in response to low ambient concentration of GABA [14]. Consistently, extrasynaptic GABA_A_Rs generally display higher affinity for GABA, slower desensitization, and lower conductance, with respect to synaptic receptors [15, 16]. GABA_A_Rs are composed of the assembly of 5 of 19 available subunits, generally two alpha (alpha 1 to 6), two beta (beta 1 to 3), and either one gamma or delta subunits [17]. As a general rule, the gamma subunit is necessary for the synaptic localization, while delta-containing receptors are excluded from synapses [18, 19]. However, the composition of extrasynaptic GABA_A_Rs that mediate tonic currents appears to be quite heterogeneous. There are evidences supporting the contribution to tonic transmission of both delta and gamma subunits, and of all of the five different alpha subunits expressed in the hippocampus (alpha 1 to 5) [20–22].

We previously demonstrated that PCDH19 downregulation in hippocampal neurons impaired GABA_A_R receptor (GABA_A_R) surface expression and miniature inhibitory postsynaptic currents (mIPSCs) [10]. However, whether PCDH19 might affect GABA_A_R-mediated tonic current in addition to mIPSCs is unknown. This is a relevant question, since tonic transmission drives the migration and morphological maturation of neurons in the developing brain and is a key determinant of neuronal excitability in the adult brain [23–25]. More in general, tonic inhibition, by shaping neuronal network activity, is involved in epilepsy, sleep, and cognitive processes [22].

Likewise, PCDH19-dependent GABA_A_R modulatory mechanisms other than receptor surface availability remain unexplored. Indeed, GABA_A_R-mediated transmission depends both on the number of cell-surface receptors and on their biophysical properties, *in primis* channel conductance and kinetics [14, 18, 26–28]. Subunit composition, allosteric modulators, and interacting proteins all contribute to the shaping of GABA_A_R biophysical properties [26, 29–33].

Here, we provide an expanded view of the GABA_A_R binding-protein PCDH19 and its modulatory role on GABAergic transmission. Our findings unveil that the regulation of GABAergic transmission by PCDH19 is not restricted to phasic currents but extends to the tonic component. Moreover, PCDH19 emerges as a multitasking GABA_A_R binding-partner that, besides regulating GABA_A_R surface expression [10], also regulates channel gating, with crucial implication for neuronal excitability and EIEE9 pathogenesis.

## Materials and Methods

### Neuronal Cultures and Transfection

**Fig. 1:**
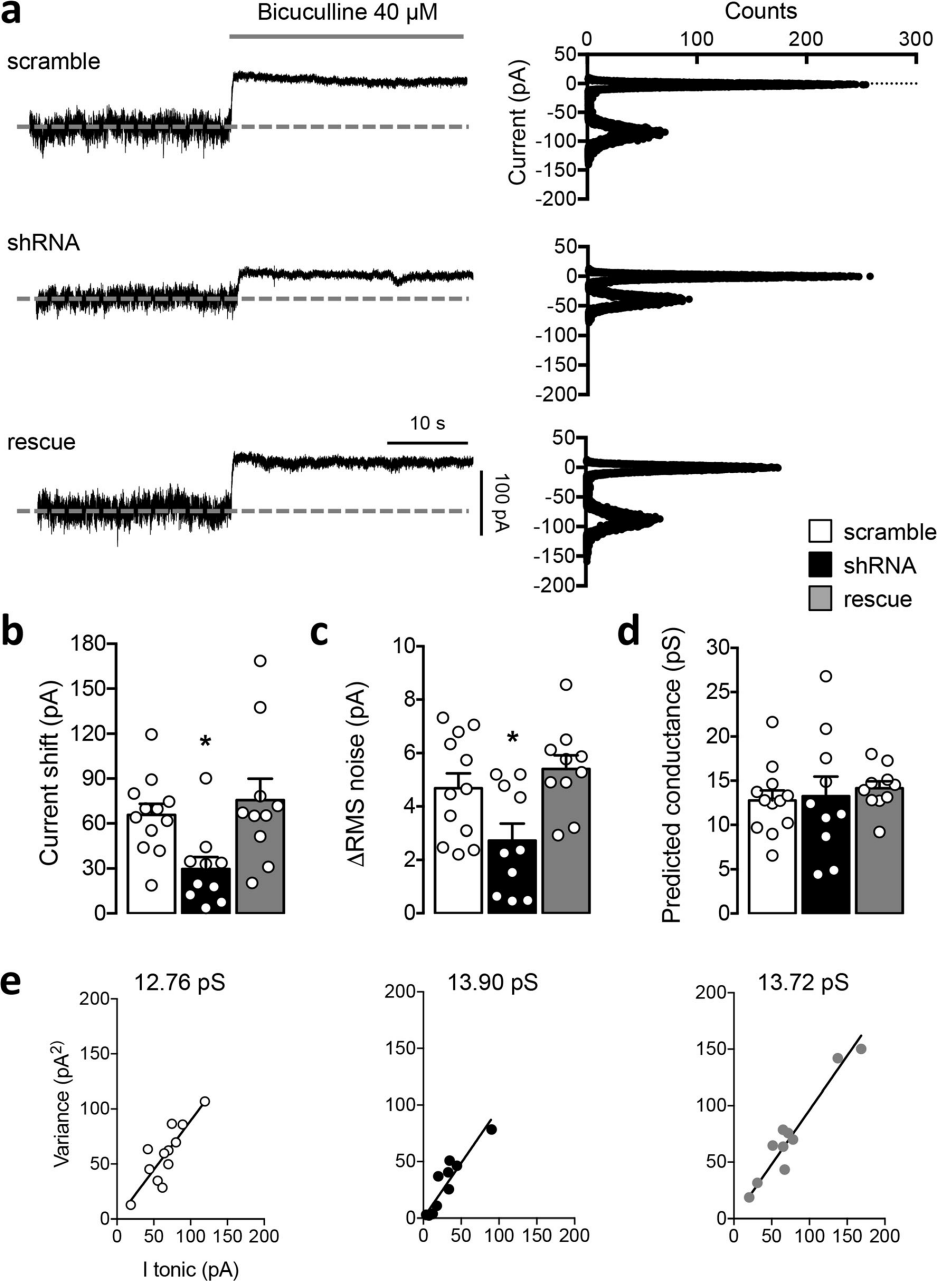
PCDH19 shRNA-mediated downregulation reduces the GABA tonic current in hippocampal neurons. **a** Left, representative current traces recorded from neurons expressing control shRNA (scramble), PCDH19 shRNA (shRNA), and shRNA + PCDH19 (rescue), showing the block of GABA_A_R tonic current upon bicuculline application (40 μM, gray bar), as inferred from the outward shift in the holding current. The dashed line indicates the holding current at baseline level, in the presence of GABA 0.1 μM. Right, corresponding all-points histograms before (baseline) and during bicuculline application. The mean current values of the two conditions, corresponding to the peaks of the Gaussian distribution, were used to calculate the current shift shown in **b**. **b** Histograms showing mean tonic current amplitude, calculated as the difference in the holding current before and during bicuculline application (bicuculline–baseline). ShRNA reduced tonic current by 55.8% with respect to scramble, while the rescue condition restored the tonic current to 115% of its control value (current shift amplitude, pA: scramble 65.731 ± 7.351, shRNA 29.707 ± 7.923, rescue 75.634 ± 14.305; one-way ANOVA, *F* (2, 29) = 5.496, *p* = 0.009; Dunnett’s post hoc test: scramble vs shRNA, *p = 0.029, DF = 29; scramble vs rescue, n.s., DF = 29; *N* = 10–12 neurons per condition from 3 different cultures). Error bars are mean ± SEM. **c** Histograms showing the current noise in scramble, shRNA, and rescue neurons expressed as the difference between the RMS noise in the baseline and the bicuculline condition. The RMS noise change was lower in shRNA compared to the other conditions (ΔRMS noise, pA: scramble 4.681 ± 0.557, shRNA 2.726 ± 0.627, rescue 5.405 ± 0.512; one-way ANOVA, *F* (2, 29) = 5.622, *p* = 0.008; Dunnett’s post hoc test: scramble vs shRNA, *p = 0.037, DF = 29; scramble vs rescue, n.s. *p* = 0.568, DF = 29; *N* = 10–12 neurons per condition from 3 different cultures). Error bars are mean ± SEM. **d, e** Prediction of GABA_A_R conductance based on noise variance analysis. Unitary channel conductance was estimated from the ratio between noise variance and mean tonic current (**d**) or from the slope of the lines representing the linear regression equation of data points obtained by plotting variance values against tonic current (**e**). No significant changes were observed in channel conductance. **d** Predicted single-channel conductance estimated from the ratio between noise variance and mean tonic current, pS: scramble 12.773 ± 1.243, shRNA 13.253 ± 2.216, rescue 14.149 ± 0.785; one-way ANOVA, *F* (2, 29) = 0.228, *p* = 0.797; *N* = 10–12 neurons per condition from 3 different cultures. **e** Predicted single-channel conductance estimated from the slope of the *I* tonic/variance linear regression equation, pS: scramble 12.76, shRNA 13.90, rescue 13.72; *N* = 9–12 neurons per condition from 3 different cultures. Slopes of the three regression lines were compared using one-way ANOVA (*F* (2,25) = 0.016, *p* = 0.983). Error bars are mean ± SEM

**Fig. 2:**
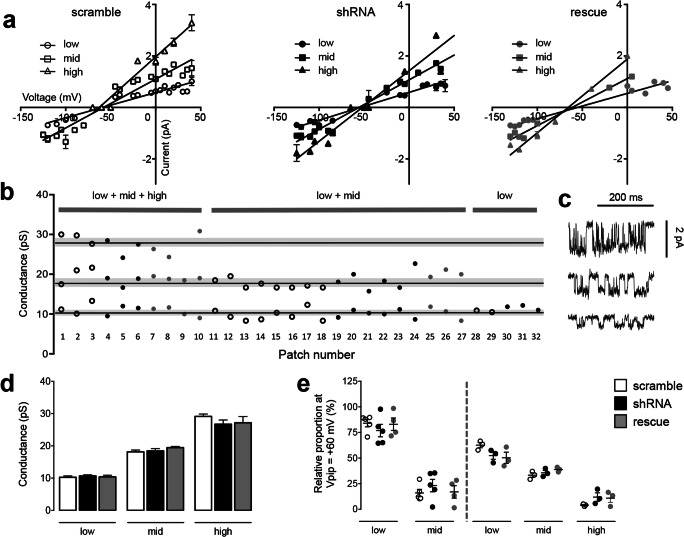
GABA_A_R single-channel conductance states are unaffected by PCDH19 downregulation. **a** *I*/*V* plots from somatic membrane patches of neurons at DIV11–15, previously transfected at DIV4 with scramble, shRNA, or rescue constructs, as indicated. Three different conductance states (low, mid, and high) are detectable in every condition, as can be inferred from the different slope of *I*/*V* curves, obtained from the linear regression equation of *I*/*V* values (*N* = 7–13 patches per condition from 3–5 independent cultures). Error bars are mean ± SEM. **b** Conductance states recorded from each patch. Patches were recorded from neurons transfected with scramble (white dots), shRNA (black dots), or rescue (gray dots). Patches can be classified according to the conductance states observed: most patches (56.3%) display low- and mid-conductance states, some patches (28.1%) display all three conductance states, and few patches (15.6%) display exclusively the low-conductance state. Solid lines indicate the mean conductance, while gray shadows indicate SEM (low conductance: 10.455 ± 0.233, *N* = 32; mid conductance: 18.596 ± 0.337, *N* = 27; high conductance 27.675 ± 0.797, *N* = 9; *N* indicates patches from three different conditions, 3–5 different cultures). **c** Representative single-channel openings from a control neuron (scramble condition), showing openings of different conductance states (from top to bottom: high, mid, and low). **d** Mean conductance values characterizing low, mid, and high conductance states for scramble (white bars), shRNA (black bars), and rescue (gray bars) neurons. PCDH19 downregulation does not affect single-channel conductance values (low conductance, pS: scramble 10.268 ± 0.416, *N* = 13; shRNA 10.688 ± 0.335, *N* = 12; rescue 10.403 ± 0.511, *N* = 7; one-way ANOVA, *F* (2, 29) = 0.310, *p* = 0.736; mid conductance, pS: scramble 18.154 ± 0.546, *N* = 11; shRNA 18.452 ± 0.692, *N* = 9; rescue 19.476 ± 0.337, *N* = 7; one-way ANOVA, *F* (2, 24) = 1.291, *p* = 0.293; high conductance, pS: scramble 29.14 ± 0.739, *N* = 3; shRNA 26.722 ± 1.31, *N* = 3; rescue 27.163 ± 1.922, *N* = 3; one-way ANOVA, *F* (2, 6) = 0.835, *p* = 0.479; *N* indicates number of patches, each patch coming from a distinct cell from 3–5 independent cultures). Error bars are mean ± SEM. **e** Relative proportion of conductance states within a single patch, showing no difference between scramble, shRNA, and rescue neurons. Patches showing two conductance states (low and mid) are plotted on the left side and analyzed separately from patches showing three conductance states (low, mid, and high) and plotted on the right (patches showing low + mid conductance states: low conductance, %: scramble 84.062 ± 3.578, *N* = 5; shRNA 76.855 ± 6.093, *N* = 5; rescue 83.021 ± 6.059, *N* = 4; one-way ANOVA, *F* (2, 11) = 0.568, *p* = 0.583; mid conductance, %: scramble 15.942 ± 3.578, *N* = 5; shRNA 23.145 ± 6.093, *N* = 5; rescue 16.979 ± 6.059, *N* = 4; one-way ANOVA, *F* (2, 11) = 0.568, *p* = 0.583; patches showing low + mid + high conductance states: low conductance, %: scramble 62.64 ± 2.541, *N* = 3; shRNA 52.437 ± 3.628, *N* = 3; rescue 50.285 ± 5.477, *N* = 3; one-way ANOVA, *F* (2, 6) = 2.635, *p* = 0.151; mid conductance, %: scramble 33.295 ± 2.222, *N* = 3; shRNA 35.624 ± 2.295, *N* = 3; rescue 38.810 ± 1.337, *N* = 3; one-way ANOVA, *F* (2, 6) = 1.917, *p* = 0.227; high conductance, %: scramble 4.062 ± 0.67, *N* = 3; shRNA 11.94 ± 4.119, *N* = 3; rescue 10.905 ± 4.171, *N* = 3; one-way ANOVA, *F* (2, 6) = 1.589, *p* = 0.281; *N* indicates number of patches, each patch coming from a distinct cell, from 3–5 independent preparations). Error bars are mean ± SEM

**Fig. 3:**
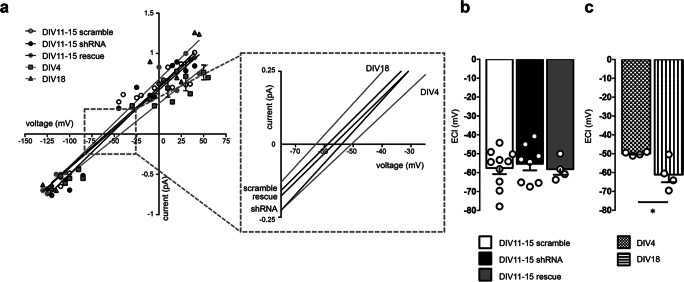
PCDH19 shRNA-mediated downregulation between DIV4 and DIV11–15 does not affect chloride reversal potential (*ECl*) in hippocampal neurons. **a** *I*/*V* plots from neurons at DIV4, DIV11–15, and DIV18. DIV4 and DIV18 neurons were untransfected, while DIV11–15 neurons had been transfected at DIV4 with scramble, shRNA, or rescue constructs. Solid lines represent fits of the linear regression equation. The inset shows magnification of line intersections with different membrane potential values, corresponding to *ECl* (*N* = 4–10 neurons per condition from 3 to 5 different cultures). Error bars are mean ± SEM. **b** Histogram showing *ECl* obtained by linear fitting of *I*/*V* plots from neurons at DIV11–15 transfected with scramble, shRNA, and rescue as indicated. PCDH19 downregulation does not change *ECl* value (*ECl* at DIV11–15, mV: scramble − 57.554 ± 3.257, *N* = 10; shRNA − 55.294 ± 3.504, *N* = 8; rescue − 58.178 ± 2.954, *N* = 4; one-way ANOVA, *F* (2, 19) = 0.171, *p* = 0.844; *N* indicates number of patches, each patch coming from a distinct cell from 3 to 5 different cultures). Error bars are mean ± SEM. **c** Histogram showing *ECl* obtained by linear fitting of *I*/*V* plots from untrasfected neurons at DIV4 and DIV18. The *ECl* is lower in DIV4 neurons compared to DIV18 neurons (*ECl*, mV: DIV4 − 49.95 ± 0.473, *N* = 4; DIV18 − 61.11 ± 4.033, *N* = 4; two-tailed unpaired Student’s *t* test, *t* = 2.748, DF = 6, *p = 0.033; *N* indicates number of patches, each patch coming from a distinct cell). Error bars are mean ± SEM

**Fig. 4:**
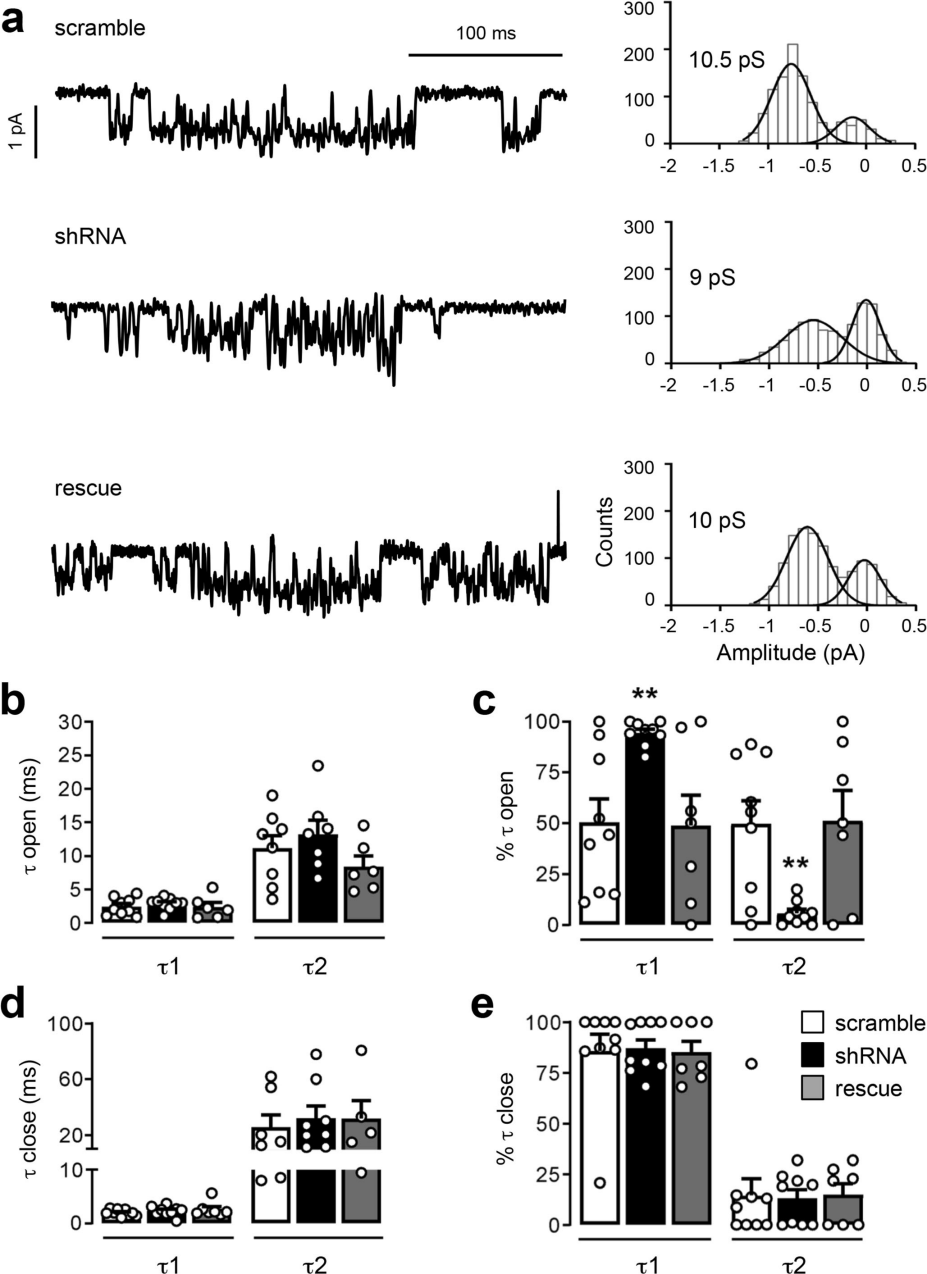
PCDH19 downregulation affects GABA_A_R kinetics. **a** Representative current traces of GABA_A_R single-channel low-conductance recordings evoked by 100 μM GABA (left) and relative current amplitude histograms fitted with Gaussian functions (right) in scramble, PCDH19 shRNA, and rescue hippocampal neurons. **b**–**e** Kinetic parameters of single-channel currents from neurons as in **a**. Time constants (**b**, **d**) and relative proportions (**c**, **e**) of the two exponential components (*τ*1 and *τ*2) that best represent the distribution of openings (**b**, **c**) and closures (**d**, **e**). In PCDH19 shRNA-expressing neurons, the relative contribution of short openings increases at the expense of that of long openings. Total number of patches recorded: scramble *N* = 9; shRNA *N* = 9; rescue *N* = 7. **b** *τ*1 open, ms: scramble 2.421 ± 0.433, *N* = 9; shRNA 2.860 ± 0.293, *N* = 9; rescue 2.353 ± 0.686, *N* = 6; one-way ANOVA *F* (2, 21) = 0.385, *p* = 0.685; *τ*2 open, ms: scramble 11.138 ± 1.893, *N* = 8; shRNA 13.221 ± 2.081, *N* = 7; rescue 8.422 ± 1.539, *N* = 6; one-way ANOVA *F* (2, 18) = 1.478, *p* = 0.254. **c** *τ*1 open, %: scramble 50.367 ± 11.436, *N* = 9; shRNA 94.322 ± 1.941, *N* = 9; rescue 48.871 ± 14.839, *N* = 7; *τ*2 open, %: scramble 49.618 ± 11.437, *N* = 9; shRNA 5.656 ± 1.934, *N* = 9; rescue 51.20 ± 14.87, *N* = 7; two-way ANOVA considering transfection and % of *τ* open as factors; transfection: *F* (2, 44) = 1.2e−5, *p* > 0.9999; % *τ* open: *F* (1, 44) = 12.06, *p* = 0.001; interaction *F* (2, 44) = 13.33, *p* < 0.0001; Dunnett’s post hoc test: % *τ*1 open, scramble vs shRNA, **p = 0.005, DF = 44; scramble vs rescue, n.s. *p* = 0.993, DF = 44; % *τ*2 open: scramble vs shRNA, **p = 0.005, DF = 44; scramble vs rescue, n.s. *p* = 0.992, DF = 44. **d** *τ*1 close, ms: scramble 1.982 ± 0.202, *N* = 9; shRNA 2.684 ± 0.304, *N* = 9; rescue 2.583 ± 0.522, *N* = 7; one-way ANOVA, *F* (2, 22) = 0.748, *p* = 0.485; *τ*2 close, ms: scramble 25.76 ± 8.503, *N* = 7; shRNA 32.190 ± 8.505, *N* = 8, rescue 31.90 ± 12.82, *N* = 5; one-way ANOVA, *F* (2, 17) = 0.149, *p* = 0.863. **e** *τ*1 close, %: scramble 85.667 ± 8.409, *N* = 9; shRNA 87.011 ± 4.211, *N* = 9; rescue 85.171 ± 5.387, *N* = 7; *τ*2 close, %: scramble 14.333 ± 8.409, *N* = 9; shRNA 12.989 ± 4.211, *N* = 9; rescue 14.829 ± 5.387, *N* = 7; two-way ANOVA considering transfection and % of *τ* close as factors; transfection: *F* (2, 44) = − 2.11e−14, *p* > 0.999; % *τ* close: *F* (1, 44) = 184.8, *p* < 0.0001; interaction: *F* (2, 44) = 0.044, *p* = 0.958. **a**–**e** *N* indicates number of patches, each patch coming from a distinct cell from 3 to 5 different cultures; error bars are mean ± SEM

**Fig. 5:**
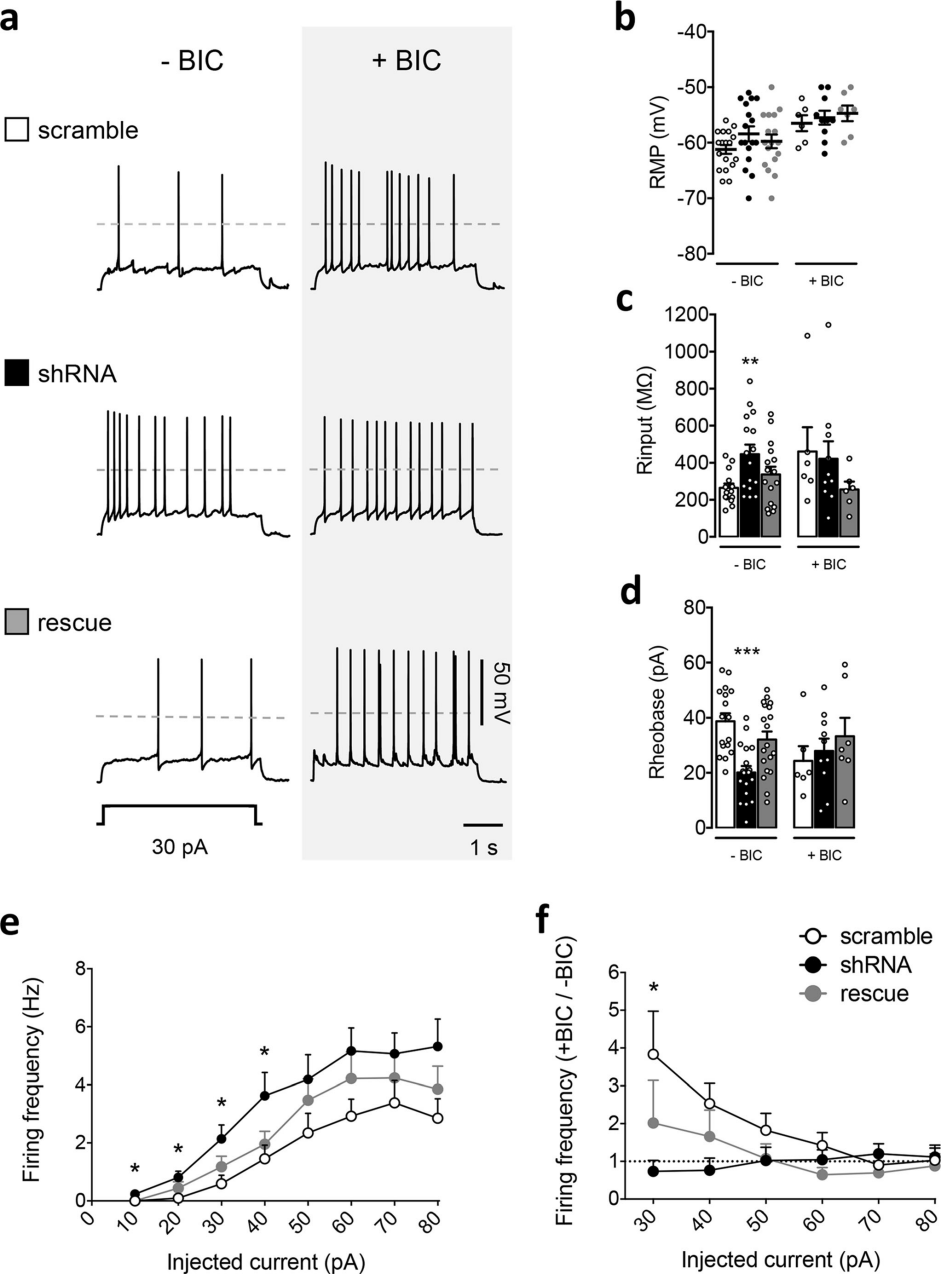
Hippocampal neurons in which PCDH19 is downregulated display increased excitability (scramble, white; shRNA, black; rescue, gray). **a** Representative traces of evoked spiking activity in hippocampal neurons expressing scramble, shRNA, and rescue constructs under basal condition (− BIC, left) and in the presence of 40 μM bicuculline (+ BIC, right). Spiking activity was evoked by 4-s squared pulse current injections (30 pA). Dotted gray lines represent 0 mV. **b** Quantification of RMP of neurons transfected as in **a**. Scramble, shRNA, and rescue neurons show no differences both under basal condition (− BIC, RMP, mV: scramble − 61.22 ± 0.802, shRNA − 58.41 ± 1.356, rescue − 59.76 ± 1.226; one-way ANOVA, *F* (2, 49) = 1.532, *p* = 0.226; *N* = 17–18 neurons per condition from 6 to 7 different cultures) and in the presence of bicuculline (+ BIC, RMP, mV: scramble − 56.50 ± 1.432, shRNA − 55.50 ± 1.258, rescue − 54.71 ± 1.409; one-way ANOVA, *F* (2, 20) = 0.358, *p* = 0.703; *N* = 6–10 neurons per condition from 3 different cultures). Error bars are mean ± SEM. **c** Histogram showing the input resistance (*R* input) measured as the slope of linear fits to the voltage responses during subthreshold current injections. Under basal condition, shRNA neurons display a significantly higher input resistance compared to scramble neurons, consistent with the decreased tonic current (− BIC , mean *R* input, mΩ: scramble 264.2 ± 23.87, shRNA 446.3 ± 50.91, rescue 336.9 ± 42.21; one-way ANOVA, *F* (2, 43) = 4.733, *p* = 0.013; Dunnett’s post hoc test: scramble vs shRNA, **p = 0.007, DF = 43; scramble vs rescue, n.s. *p* = 0.379, DF = 43; *N* = 14–16 neurons per condition from 6 to 7 different cultures). In the presence of bicuculline, no differences were observed between the three groups of neurons (+ BIC, mean *R* input, mΩ: scramble 461.3 ± 130.2, shRNA 421.8 ± 93.26, rescue 255.7 ± 43.05; one-way ANOVA, *F* (2, 19) = 1.049, *p* = 0.369; *N* = 6–10 neurons per condition from 3 different cultures). Error bars are mean ± SEM. **d** Quantification of rheobase current from neurons transfected as in **a**. Under basal condition, shRNA-expressing neurons, contrary to rescue neurons, are characterized by a reduced rheobase compared to scramble (− BIC, rheobase, pA: scramble 38.77 ± 2.879, shRNA 20.11 ± 2.374, rescue 32.10 ± 2.903; one-way ANOVA, *F* (2, 51) = 11.70, *p* < 0.0001; Dunnett’s post hoc test: scramble vs shRNA, ***p < 0.0001, DF = 51; scramble vs rescue, n.s. *p* = 0.159, DF = 51; *N* = 17–19 neurons per condition from 6 to 7 different cultures). In the presence of bicuculline, no differences were observed between the three groups of neurons (+ BIC, rheobase, pA: scramble 24.36 ± 5.310, shRNA 27.96 ± 4.469, rescue 33.30 ± 6.712; one-way ANOVA, *F* (2, 20) = 0.588, *p* = 0.564; *N* = 6–10 neurons per condition from 3 different cultures). Error bars are mean ± SEM. **e** Relationship between injected current and firing frequency of neurons transfected as in **a** under basal condition. Spiking activity was evoked by current steps starting from 0 pA up to 80 pA, in 10-pA increments. shRNA-expressing neurons, contrary to rescue neurons, display higher firing frequency compared to control neurons. Statistical significance is reached between 10 and 40 pA, and a trend was observed for the other current steps (firing frequency at 10 pA, Hz: scramble 0.000 ± 0.000, shRNA 0.234 ± 0.117, rescue 0.000 ± 0.000; one-way ANOVA, *F* (2, 46) = 4.115, *p* = 0.027; Dunnett’s post hoc test: scramble vs shRNA, *p = 0.0321, DF = 46; scramble vs rescue, n.s. *p* > 0.999, DF = 46; firing frequency at 20 pA, Hz: scramble 0.093 ± 0.093, shRNA 0.812 ± 0.217, rescue 0.441 ± 0.219; one-way ANOVA, *F* (2, 46) = 3.590, *p* = 0.035; Dunnett’s post hoc test: scramble vs shRNA, *p = 0.019, DF = 46; scramble vs rescue, n.s. *p* = 0.325, DF = 46; firing frequency at 30 pA, Hz: scramble 0.593 ± 0.292, shRNA 2.141 ± 0.480, rescue 1.176 ± 0.366; one-way ANOVA, *F* (2, 46) = 3.998, *p* = 0.025; Dunnett’s post hoc test: scramble vs shRNA, *p = 0.014, DF = 46; scramble vs rescue, n.s. *p* = 0.463, DF = 46; firing frequency at 40 pA, Hz: scramble 1.453 ± 0.470, shRNA 3.625 ± 0.8, rescue 1.956 ± 0.449; one-way ANOVA, *F* (2, 46) = 3.647, *p* = 0.033; Dunnett’s post hoc test: scramble vs shRNA, *p = 0.024, DF = 46; scramble vs rescue, n.s. *p* = 0.770, DF = 46; *N* = 14–17 neurons per condition from 6 to 7 different cultures). Error bars are mean ± SEM. **f** Firing frequency ratio between bicuculline and basal condition (+ BIC/− BIC) of neurons transfected as in **a**. ShRNA-expressing neurons, contrary to rescue neurons, display a lower firing frequency ratio compared to control neurons. Statistical significance is reached at 30 pA, and a trend was observed between 40 and 60 pA (firing frequency ratio at 30 pA: scramble 3.833 ± 1.144, shRNA 0.734 ± 0.292, rescue 2.017 ± 1.127; one-way ANOVA, *F* (2, 20) = 3.598, *p* = 0.046; Dunnett’s post hoc test: scramble vs shRNA, *p = 0.026, DF = 20; scramble vs rescue, n.s. *p* = 0.269, DF = 20; *N* = 6–10 neurons per condition from 3 different cultures). Error bars are mean ± SEM

For primary neuronal rat cultures, Sprague Dawley timed-pregnant adult rats were purchased from Charles River Laboratories (Italy). Hippocampal neurons were prepared from rat embryos of either sex at embryonic day (E)18 as previously described [34]. The neurons were plated on coverslips coated with poly-d-lysine in multiwell culture plates in a 12-well format, at a density of 75,000/well. The neurons were grown in Neurobasal Medium (Life Technologies, Italy) supplemented with homemade B-27, 0.25% l-glutamine, 1% penicillin/streptomycin, and 0.125% glutamate (Sigma Aldrich) and maintained at 37 °C, 5% CO_2_. The B-27 was prepared as previously described [35], except for a final medium concentration of 2.5 μg/ml of apo-transferrin (Sigma-Aldrich, Italy) instead of 5 μg/ml of holo-transferrin. Hippocampal neurons at days in vitro (DIV) 4 were transfected with standard calcium phosphate method and pyramidal cells were analyzed at DIV13–15 (Figs. 1 and 5; Supplementary Figure S1 and S2) or DIV11–15 (Figs. 2, 3, and 4). All animal care and experimental procedures were performed in accordance with the CNR licensing and were approved by the Italian Ministry of Health (authorization no. 100/2016 and 2D46A.N.463).

### cDNA and shRNA Constructs

All plasmids used to transfect neurons were previously reported in the Bassani et al. paper, in which the validation by western blotting of PCDH19 shRNA and rescue strategy was also provided [10]. Briefly, the *PCDH19* open reading frame with a C-terminal V5 tag (human isoform 4, lacking amino acid 892 of the canonical isoform, accession number NM_001184880.1; gift from Prof. J. Gecz, University of Adelaide) was cloned into cFUW (Addgene). PCDH19 short hairpin (sh)RNA (target sequence: 5′-gagcagcatgaccaatacaat-3′) or control shRNA (scramble, target sequence: 5′-gctgagcgaaggagagat-3′) were cloned into the pLVTHM vector (Addgene). The shRNA target sequence has 100% homology with the rat *Pcdh19* sequence (NM_001169129.1), while it harbors two mismatches (5′-gagcagca*c*gaccaatacaa*c*-3′, mismatches in italics) with the human *PCDH19* sequence (NM_001184880.1), conferring PCDH19-V5 partial resistance to the shRNA. Hence, PCDH19-V5 and shRNA were used in combination for the rescue experiments.

### Immunocytochemistry, Image Acquisition, and Analysis

For the immunocytochemistry (ICC) experiments, hippocampal neurons were fixed at DIV13 with 4% paraformaldehyde + 4% sucrose for 8 min at room temperature. The neurons were incubated with primary (rabbit anti-PCDH19, 1:400, Bethyl Laboratories, USA) and secondary antibody (rabbit Alexa fluor 555, 1:300, Thermo Fisher Scientific, Italy) in gelatin detergent buffer (GDB: 30 mM phosphate buffer at pH 7.4 containing 0.2% gelatin, 0.5% Triton X-100, and 0.8 M NaCl) overnight at 4 °C and for 1 h at room temperature, respectively.

Images were acquired with a Zeiss LSM 800 confocal microscope (Carl Zeiss, Italy) by using × 63/1.4 oil objective. The transfected neurons were identified based on the presence of GFP encoded by the pLVTHM vector, and images were obtained from the z-projection (maximum intensity) of 3–4 stacks at 0.60-μm intervals (1024 × 1024 pixel resolution). The images were analyzed with Fiji software: a mask was created on the GFP channel to select neuronal dendrites and the mean intensity of PCDH19 was measured.

### Electrophysiological Recordings

Patch-clamp recordings were collected using an Axopatch 200B amplifier (Molecular Device, CA, USA). Data were digitized at 5 kHz and filtered at 1 kHz with a Digidata 1322 acquisition system. The acquisition software used was Clampex 9.2 (Axon Instruments, Inc., CA, USA). Patch pipettes (GB150F-8P with filament, Science Products) were pulled from hard borosilicate glass on a Brown-Flaming P-87 puller (Sutter Instruments, Novato, CA, USA) and fire-polished to a final electrical resistance of 5–7 MΩ for whole-cell recordings and 8–15 MΩ for cell-attached recordings. All the experiments were performed at room temperature on dissociated hippocampal pyramidal neurons maintained in a solution containing (in mM) 138 NaCl, 4 KCl, 2 CaCl_2_, 1.2 MgCl_2_, 10 HEPES, and 10 d-glucose, adjusted to pH 7.4 with NaOH. For experiments in whole-cell configuration, series resistance was monitored and, if it was > 20 MΩ, the recording was discarded. Data analysis was performed with Clampfit 10.3 (Molecular Devices, CA, USA).

#### Tonic Current Measurement and Analysis

GABA_A_R-mediated tonic currents were recorded in whole-cell voltage-clamp mode at a holding potential (Vh) of − 70 mV in the presence of kynurenic acid (3 mM) to block glutamatergic transmission and GABA (0.1 μM) to increase the amplitude of tonic current and reduce variability among cultures. Pipettes were filled with a Cs-based solution containing (in mM) 140 CsCl, 1 MgCl_2_, 1 CaCl_2_, 10 EGTA, 10 HEPES, and 0.1 GTP-Na^+^ (adjusted to pH 7.4 with CsOH) supplemented with the Na^+^ channel blocker QX 314 bromide (4 mM). The amplitude of GABA_A_R-mediated tonic current was calculated by the outward shift in the baseline current induced by the GABA_A_R antagonist bicuculline (40 μM) [16, 28, 36, 37]. After obtaining a stable recording for at least 1 min (untreated control condition), bicuculline was applied by using a gravity-driven perfusion system (RSC-200, BioLogic, France) through a micropipette positioned close to the soma of the recorded cell. All-points histograms were generated from 60-s epochs relative to untreated and bicuculline conditions. Tonic currents were defined by fitting Gaussian curves to these histograms. The peak of the Gaussian distribution (*μ*) represents the mean holding current, while the standard deviation of the curve (*σ*) represents the root mean square (RMS) of the variance over the 60-s interval. Tonic current was calculated both as the difference between the *μ* values relative to untreated and bicuculline epochs (current shift, *I*_mean_) and difference of RMS noise between the two conditions [28]. Changes in the RMS noise values before and during bicuculline application were also used to predict the single-channel conductance (*γ*) of tonic receptors. First, the squared standard deviation of the curve (*σ*^2^) was plotted against the *I*_mean_ to extrapolate the single-channel current (*i*), based on the parabolic relationship described by the following formula: *σ*^2^(*I*_mean_) *= i*(1 − *P*_O_)*I*_mean_, where *P*_O_ is the channel open probability. In our experimental conditions, the *P*_O_ of the GABA_A_R mediating tonic current is expected to be small, as the exogenous GABA concentration is low [36]. Therefore, the relationship between *σ*^2^ and *I*_mean_ can be well approximated by the following equation: *i* = *σ*^2^/*I*_mean_ [16, 36]. Next, single-channel conductance was calculated using the equation *γ* = *i*/(*Vh* − *ECl*), where *Vh* is the holding potential and *ECl* is the chloride electrochemical equilibrium potential (or reversal potential), corresponding to approximately 0 mV in our experimental conditions.

#### Cell-Attached Recordings

GABA_A_R single-channel currents were recorded in voltage-clamp mode, cell-attached configuration. Patch pipettes were filled with the extracellular solution (composition described above) supplemented with 100 μM 4,4′-diisothiocyanato-2,2′-stilbenedisulfonic acid disodium salt (DIDS) to block ClC type Cl^−^ channel activity, 1 mM 4-aminopyridine (4-AP), and 5 mM tetraethylammonium chloride (TEA-Cl) to block K^+^ channel activity, and 100 μM GABA to evoke GABA_A_R activity. Following the formation of a giga seal (> 10 GΩ), the output gain was set to 100 mV/pA and the headstage switched to the capacitive feedback mode. At the end of each single-channel recording, the patch membrane was ruptured to measure the resting membrane potential (RMP) of the cell. Although the intrapipette solution was not suitable for whole-cell experiments, RMP values, measured immediately after the rupture of the membrane, were analogous to those obtained in whole-cell experiments using a K-gluconate-based internal solution (values in Fig. 5). GABA_A_R single-channel currents were elicited by voltage steps ranging from − 60 to +100 mV (corresponding to a patch pipette voltage (Vpip) ranging from − 100 to + 60 mV) in 20-mV increments and 800-ms duration. For each patch, at each voltage step, all the sweeps where the channel opened were concatenated and analyzed. All-points histograms were generated from single-channel openings at each potential and fitted with the Gaussian equation. The peak of the Gaussian distribution represents the mean single-channel current, which was plotted against voltage to obtain the *I*/*V* relationship. Voltages were previously adjusted according to the measured RMP for each cell. The point at which the *I*/*V* curve crosses the voltage axis represents the reversal potential of the GABA_A_R-mediated current, which corresponds to the chloride reversal potential (*ECl*). Single-channel slope conductance for an individual cell was calculated from the slope of the linear regression obtained from the *I*/*V* plots. For those cells in which slope conductance could not be obtained, the chord conductance (*γ*_chord_) at single potential was calculated according to the equation *γ*_chord_ = *i*/(*Vh* − *ECl*) where *i* is the observed single-channel current, *Vh* the holding potential, and *ECl* the chloride reversal potential. Open and close times were analyzed at Vpip = + 60 mV, corresponding to a Vh approximately 60 mV below the *ECl*, which is the same condition used to measure whole-cell tonic current.

Duration histograms were fitted with a mixture of exponential distributions defined by

$$ f(t)=\sum \limits_{i=1}^n\left(\frac{a_i}{\tau_i}\right)\exp \left(-\frac{t}{\tau_i}\right) $$

and characterized by a time constant *τ* and a relative area *a*. Since concatenated traces were analyzed, we reported only the short close times that likely characterize intra-cluster shutting events, and thus likely define the kinetic of a single channel.

#### Current-Clamp Recordings

The intrinsic excitability of neurons was evaluated by whole-cell recordings. To elicit action potentials (APs), neurons were held at − 60 mV and stimulated with depolarizing current injections of 4 s of duration and amplitude ranging from 0 to + 80 pA (current steps, Δ*I* = 10 pA). Patch pipettes were filled with a solution containing (in mM) 126 K-gluconate, 4 NaCl, 0.05 CaCl_2_, 0.1 EGTA, 10 HEPES, 10 d-glucose, 1 MgCl_2_, 3 ATP-Mg^2+^, and 0.1 GTP-Na^+^, adjusted to pH 7.2 with KOH. Bath application of bicuculline (40 μM) was used to block GABA_A_R currents. To assess individual action potential (AP) waveforms, the first spike evoked by the minimum amount of current injected (rheobase) was analyzed. Spike width was measured at 50% of the peak amplitude, which was calculated from the AP voltage threshold to the peak of APs. To extrapolate the AP voltage threshold values, phase-plane plots were constructed from the first time derivative of voltage (dV/dt) plotted against the membrane voltage [38]. AP threshold was defined as the voltage value at which dV/dt was 4% of its maximal value (dV/dt_max_) [39, 40]. Rheobase was calculated by measuring the minimum amount of current injection able to induce a spike from Vh = − 60 mV. Input resistance (*R* input) was calculated as the slope of the *V*/*I* relationship obtained by plotting the steady-state membrane depolarization elicited by a series of subthreshold current steps of 1 pA increment and 4 s of duration.

### Statistical Analysis

Three neuronal groups were compared: neurons transfected with scramble, shRNA, and rescue constructs. Statistical significance of the data was assessed by one-way ANOVA, considering transfection condition as the main factor. Two factors were considered for the two-way ANOVA test used to analyze data presented in Fig. 4c, e (transfection and % of *τ* open or *τ* close). ANOVA tests were followed by Holm-Sidak’s (Supplementary Figure S1) or Dunnett’s (all other figures) multiple-comparison post hoc test. Two-tailed unpaired Student’s *t* test was used to assess the statistical significance of the pairwise comparison shown in Fig. 3c. GraphPad Prism (version 6, GraphPad Software) was used for plotting data and for statistical analyses. All data are mean ± SEM. The threshold for statistical significance was *p* < 0.05. Asterisks indicate a *p* value < 0.05, and nonsignificance is denoted by “n.s.” Detailed statistical results, including exact *p* values, *F* factor, and degrees of freedom (DF), as well as cell number (*N*), are provided in the figure legends. Neurons were obtained from the following number of independent preparations: 3 cultures for experiments of Fig. 1 and Supplementary Figure S1; 3–4 cultures for experiments of Figure S2; 3–5 cultures for experiments of Figs. 2, 3, and 4; and 3–7 cultures for experiments of Fig. 5.

## Results

### PCDH19 Downregulation Reduces the GABA_A_R-Mediated Tonic Current in Hippocampal Neurons

As the first step of our study, we assessed whether PCDH19 downregulation might affect the GABA_A_R-mediated tonic current in primary hippocampal neurons. The standard method consists in applying a saturating concentration of the GABA_A_R antagonist bicuculline to block GABA_A_Rs and reveal the tonic inhibitory current from the shift in the holding current and the reduction in the current noise, which depend on extrasynaptic receptors [28]. To this end, we transfected hippocampal neurons from embryonic day (E)18 rat embryos at DIV4 with either a shRNA specific for PCDH19 (shRNA), a control shRNA (scramble), or PCDH19 shRNA plus a shRNA-resistant PCDH19 cDNA (rescue condition). We verified by ICC that shRNA neurons displayed a significant downregulation of PCDH19 expression, while rescue neurons showed an overall increase of PCDH19 dendritic signal compared to controls (scramble vs shRNA *p = 0.042, scramble vs rescue *p = 0.031; shRNA vs rescue ***p < 0.001; one-way ANOVA with Holm-Sidak’s post hoc test; Supplementary Figure S1a and S1b). At DIV13–15, we performed whole-cell patch-clamp recordings on transfected neurons. GABA (0.1 μM) was added to the extracellular solution in order to avoid fluctuations in the ambient concentration of GABA [36]. Notably, the shRNA expression reduced the current shift upon bicuculline application (40 μM) by more than 50% compared to scramble, and PCDH19 overexpression was able to completely rescue the current shift (scramble vs shRNA, *p = 0.029; scramble vs rescue, n.s.; one-way ANOVA with Dunnett’s post hoc test; Fig. 1a, b). The analysis of the current noise, quantified as the root mean square (RMS) of variance, which is the most accurate method to detect small tonic currents [28, 41], confirmed the reduction of tonic current in shRNA-transfected neurons compared to scramble. In rescue condition, the difference in the RMS noise before and during bicuculline application was fully restored (scramble vs shRNA, *p = 0.037; scramble vs rescue, n.s.; one-way ANOVA with Dunnett’s post hoc test; Fig. 1a, left panel, and c).

Altogether, these results indicate that PCDH19 downregulation leads to a reduction of GABAergic tonic current. Reduced GABAergic tone might be the result of a reduced number of GABA_A_Rs on the cell surface, changes in receptor kinetics, or both.

### PCDH19 Regulates the Kinetics of GABA_A_Rs

Changes in receptor number and kinetics both contribute to set the inhibitory tone. We recently demonstrated that PCDH19 downregulation reduces GABA_A_R surface expression in hippocampal neurons [10]. Here, we sought to determine whether PCDH19 might also regulate the biophysical properties of GABA_A_Rs.

First insights on single-channel conductance were obtained by the analysis of noise variance [16, 28, 36], as described in the methodological section. The predicted unitary current was consistent with the conductance ranges reported for receptors that mediate tonic inhibition [16, 36, 42–44], and was not statistically different for neurons transfected with either scramble, shRNA, or rescue, suggesting that PCDH19 does not regulate the conductance of GABA_A_Rs (Fig. 1d, e).

To confirm these data and study whether PCDH19 might modulate the kinetics of GABA_A_Rs, we shifted from whole-cell to cell-attached patch-clamp configuration, and recorded GABA_A_R current from single channels on the somata of neurons. Here, the chance to record currents from extrasynaptic receptors, including those that mediate tonic current, is high, since synaptic receptors are incorporated in clusters that occupy less than 1% (0.72%) of the soma surface [45].

Hippocampal neurons were transfected with shRNA, scramble, and shRNA + PCDH19 (rescue) at DIV4, and single channels were recorded at DIV11–15. We used a high concentration of GABA in the patch pipette (100 μM GABA), which is able to rapidly induce GABA_A_R desensitization and causes extrasynaptic receptors to enter prolonged closed states interrupted by discrete clusters of openings. Each cluster is supposed to derive from the repeated activation of a single channel [44].

Irrespective of transfected constructs, recorded GABA_A_Rs exhibited three distinct conductance levels, as previously reported in literature [44, 46]. In particular, low-, mid-, and high-conductance states were observed during single-patch recordings, alone or in combination (Fig. 2a–c). The average values of the conductance states were not significantly different between scramble, shRNA, and rescue neurons (Fig. 2d).

The relative percentage of conductance levels did not vary between the different conditions (scramble, shRNA, and rescue) and was characterized by a predominance of a low- followed by a mid-conductance state, while a high-conductance state, when displayed, was scarcely represented (Fig. 2e).

In almost all the cells from the three conditions recorded, the GABA_A_Rs opened predominately at the low-conductance state (Fig. 2b), which is generally attributed to GABA_A_Rs mediating tonic current [16, 36, 42], suggesting that the majority of channels recorded in single-channel experiments were indeed extrasynaptic GABA_A_Rs mediating tonic currents.

Irrespective of the transfected constructs, and as expected for GABA_A_R-mediated currents, the current/voltage (*I*/*V*) relationships of the three groups of neurons revealed a chloride reversal potential (*ECl*) close to − 60 mV, indicating a near completion of *ECl* shift towards more negative potentials by this time (Fig. 3a, b), consistent with the literature [47, 48]. The well-documented shift of *ECl* from depolarized to hyperpolarized values in neurons is attributed to the regulated expression of chloride transporters during development [49], and an *ECl* shift was detected also in our cultures (DIV4 vs DIV18 neurons, *p = 0.033; two-tailed unpaired *t* test; Fig. 3a, c). These data indicate that shRNA-mediated PCDH19 downregulation between DIV4 and DIV11–15 did not have a major effect on the modulation of *ECl*.

Next, we compared the single-channel properties of the three groups of neurons transfected at DIV4 and recorded at DIV11–15 (Fig. 4). We focused on the low-conductance state, which was the most represented and typically attributed to tonic current mediating GABA_A_Rs, to get insights into channel kinetics following PCDH19 downregulation. In particular, we compared the open and close time properties of GABA_A_Rs in neurons expressing scramble, shRNA, and rescue constructs.

In control neurons (scramble), we observed two open time constants (open *τ*1 and *τ*2), characterized by distinct durations, which were not different from those recorded from shRNA and rescue neurons (Fig. 4a, b).

However, the open time distribution, evaluated as a percentage of the open time constant areas, was significantly different following PCDH19 downregulation. In particular, while in scramble and rescue neurons open time was equally distributed between short (*τ*1) and long (*τ*2) openings, in shRNA-expressing neurons, the short openings predominated over the long openings (% *τ*1 open: scramble vs shRNA, **p = 0.005; scramble vs rescue, n.s.; % *τ*2 open: scramble vs shRNA, **p = 0.005; scramble vs rescue, n.s.; two-way ANOVA with Dunnett’s post hoc test; Fig. 4c).

By contrast, no differences were observed between the three groups of neurons, neither in close time constants (Fig. 4d) nor in close time distribution (Fig. 4e).

The preferential opening of the channel at the shortest time constant in shRNA neurons translates into a flickering behavior of GABA_A_Rs, characterized by the rapid transition between open and closed states (Fig. 4a).

In conclusion, these data indicate that PCDH19 downregulation affects GABA_A_R gating, by increasing the contribution of brief openings at the expense of long ones, which is known to decrease the current flowing through the channel per time unit [50].

### PCDH19 Downregulation Increases Neuronal Intrinsic Excitability

The capability of PCDH19 to modulate GABA_A_R gating provides a mechanism for GABAergic tone regulation, which in turn sets neuronal excitability and hence regulates neuronal network activity [22].

To investigate whether PCDH19 downregulation might affect neuronal intrinsic excitability, hippocampal neurons were transfected as before (at DIV4) with scramble, shRNA, and rescue. At DIV13–15, whole-cell patch-clamp recordings in current-clamp mode were performed to analyze passive and active membrane properties of neurons in all conditions (Fig. 5a–f).

Despite no changes in resting membrane potential (RMP) (Fig. 5b, − BIC), PCDH19 downregulation, contrary to rescue condition, led to a significant increase in the mean input resistance with respect to control neurons (scramble vs shRNA, **p = 0.007; scramble vs rescue, n.s.; one-way ANOVA with Dunnett’s post hoc test; Fig. 5c, − BIC), consistent with the reduction in tonic GABAergic currents described above (Fig. 1a–c). As a consequence, shRNA-expressing neurons required less current injection to trigger an action potential (AP) (rheobase), compared to the control condition, indicating higher intrinsic excitability. The coexpression of shRNA together with PCDH19 (rescue) restored the rheobase to control levels, thus confirming the specificity of shRNA effects (scramble vs shRNA, ***p < 0.0001; scramble vs rescue, n.s.; one-way ANOVA with Dunnett’s post hoc test; Fig. 5d, − BIC).

As further evidence of their hyperexcitability, shRNA-expressing neurons showed a higher firing frequency with respect to controls. Statistical significance was reached between 10 and 40 pA, and a trend was observed for the other current steps (firing frequency at 10 pA, scramble vs shRNA, *p = 0.032; scramble vs rescue, n.s.; 20 pA, scramble vs shRNA, *p = 0.019; scramble vs rescue, n.s.; 30 pA, scramble vs shRNA, *p = 0.014, scramble vs rescue, n.s.; 40 pA, scramble vs shRNA, *p = 0.024; scramble vs rescue, n.s.; one-way ANOVA with Dunnett’s post hoc test, Fig. 5e and Table S1a).

The analysis of the AP waveform revealed no difference in AP amplitude or width following PCDH19 downregulation (Figure S2a, b, d), and no significant difference in voltage threshold was observed between the three groups of neurons (Figure S2c, d).

To investigate the causal link between reduced GABA_A_R-mediated inhibition and increased neuronal excitability in PCDH19 shRNA-expressing neurons, we repeated analogous experiments in the presence of bicuculline (Fig. 5a–d, + BIC; Fig. 5f; and Table S1b). If this hypothesis is true, blocking GABA_A_R-mediated currents is expected to affect the firing frequency more in controls than in shRNA neurons, thus reducing the firing frequency differences between groups. This was indeed the case (Fig. 5a, f). As expected, bicuculline bath application (40 μM) increased the firing frequency of scramble and rescue neurons with respect to the basal condition, especially at low values of current injection, as indicated by a firing frequency ratio (+ BIC/− BIC) above 1. By contrast, the effect of bicuculline on shRNA-expressing neurons was negligible, and their firing frequency ratio between bicuculline and basal conditions was significantly lower with respect to the control neurons (firing frequency ratio, + BIC/− BIC, 30 pA: scramble 3.833 ± 1.144, shRNA 0.734 ± 0.292, rescue 2.017 ± 1.127; scramble vs shRNA, *p = 0.026, scramble vs rescue, n.s.; one-way ANOVA with Dunnett’s post hoc test, Fig. 5a, f and Table S1). No significant differences in RMP were observed between the three groups of neurons (Fig. 5b, + BIC), and notably, bicuculline erased the differences in input resistance and rheobase between shRNA and scramble neurons (Fig. 5c–d, + BIC).

Altogether, these results indicate that PCDH19 downregulation enhances neuronal intrinsic excitability and suggest a causal link between reduced GABAergic transmission and hyperexcitability, with important implications for the pathogenesis of *PCDH19*-related epilepsy.

## Discussion

This study stemmed from the new recently identified interaction between PCDH19 and GABA_A_Rs [10] and sought to provide a more comprehensive understanding of PCDH19’s role in GABAergic transmission and neuronal excitability.

We first demonstrated that, in primary rat hippocampal neurons, PCDH19 downregulation reduces inhibitory tonic currents, and we next investigated both the underlying mechanisms and the consequences on neuronal excitability.

When considering the mechanisms of tonic current reduction, a first explanation might be the reduction of the GABA_A_R amount on the neuronal surface, as previously demonstrated for alpha1- and alpha2-containing receptors in PCDH19 shRNA-expressing neurons [10]. Besides evidences supporting alpha1 and alpha2 contribution to tonic transmission [21, 22, 45], it is important to note that PCDH19 is also able to associate with typically extrasynaptic GABA_A_R subunits, most likely because of PCDH19 binding-site conservation among alpha subunits [10].

Notably, we demonstrated that a second non-exclusive mechanism exists and concerns the biophysical properties of GABA_A_Rs remaining on the neuronal surface. We first analyzed single-channel conductance that reflects receptor composition in addition to its gating state, i.e., the number of GABA binding sites occupied [16, 36, 44, 51]. We demonstrated that this parameter does not change upon PCDH19 downregulation. In particular, we identified three distinct conductance states within single patches from primary hippocampal neurons, suggesting that we recorded from a heterogeneous extrasynaptic receptor pool. Although we cannot exclude that receptors mediating phasic inhibition but transiently located outside the synapses [52] might have contributed to such heterogeneity, these findings are consistent with the well-known involvement of multiple receptor types (containing both delta and gamma subunits, together with different alpha subunits) in tonic transmission [20–22]. Since PCDH19 downregulation did not affect conductance state values, numbers, or their relative proportions, this suggests the overall retention of extrasynaptic GABA_A_R pool composition.

Next, we focused on the low-conductance state, which is typical of extrasynaptic receptors mediating tonic currents [16, 36, 43, 44], and we analyzed channel kinetics. We showed that, following PCDH19 downregulation, GABA_A_Rs displayed a significant change in the relative proportion of their opening times, with short openings prevailing over the long ones. The resulting flickering behavior of GABA_A_Rs is known to reduce the current flowing through the channel per time unit [50]. Since PCDH19 directly binds GABA_A_Rs, this physical interaction might induce structural rearrangements that stabilize the channel open conformation. In support of this, PCDH19 binds a 10–amino acid sequence within the intracellular loop between the transmembrane domains (TM) 3 and TM4 of alpha subunits [10]. While the regions surrounding the TM2 are important for GABA_A_R conductance and ion selectivity [53], the TM3–TM4 loop confers gating properties to the channel [54].

Even though conductance analysis argues against major changes in the composition of the extrasynaptic GABA_A_R pool upon PCDH19 downregulation, as discussed above, we cannot completely exclude some subunit composition changes within the low-conductance receptor pool, able to selectively affect kinetics. For instance, spontaneously opening GABA_A_Rs, which do not require GABA binding to activate, display a shorter average open time and lower opening probability but similar conductance with respect to the conventional pool of GABA_A_Rs. This likely relies on their subunit composition that, however, remains elusive [55, 56].

Irrespective of their relative contribution, it is reasonable to assume that both altered kinetics and reduced expression of the GABA_A_Rs on the neuronal surface are responsible for tonic current reduction in PCDH19 shRNA-expressing neurons.

Tonic inhibition is a powerful regulator of intrinsic neuronal excitability [22, 23, 57]. Consistently, we observed a decreased rheobase together with an increased spiking frequency following PCDH19 downregulation in neurons. These data fit well with the increased input resistance of shRNA-expressing neurons. When the RMP is close to *ECl*, as in our experimental conditions, alterations in Cl^−^ currents are expected to modify the input resistance rather than RMP, thus modulating the responsiveness of neurons to synaptic stimuli. Indeed, although the RMP was not affected by PCDH19 downregulation, small amounts of current injection were more effective in changing the membrane potential, and so the intrinsic excitability, in shRNA-expressing neurons compared to the control condition.

Input resistance increases as a consequence of reduced GABAergic tone but also in response to reduced cell size [58]. Since a reduced arborization has been observed in hippocampal pyramidal neurons upon PCDH19 downregulation [10], altered neuronal morphology, which might itself arise from the GABAergic signaling impairment [25, 59], could have contributed to increased input resistance and neuronal excitability.

However, acute bath application of bicuculline was able to erase the differences in input resistance between controls and shRNA neurons, thus arguing against a major influence of neuronal arborization. Notably, bicuculline also erased the differences in rheobase between groups and had a negligible effect on the firing frequency of shRNA-expressing neurons, thus supporting the existence of a causal link between reduced GABA_A_R-mediated inhibition and increased neuronal excitability in PCDH19 shRNA-expressing neurons.

The regulation of neuronal excitability is crucial to maintain the neuronal firing rate within operational ranges to prevent saturation and to limit the number of active neurons, thus allowing a low-noise and sparse information coding system [22, 60, 61]. Indeed, reduced GABA_A_R-mediated tonic current and neuronal hyperexcitability are associated with epilepsy [23, 62–64]. Moreover, tonic inhibitory transmission is involved also in the comorbidities of epilepsy, such as cognitive and behavioral processes and sleep [22, 65–68], which are often compromised in EIEE9 patients [1–3].

By describing PCDH19 as a regulator of GABAergic tonic conductance, we provide here a functional link between PCDH19 loss of function and EIEE9 phenotypes.

A critical determinant of EIEE9 pathogenesis is thought to be the cell interference: EIEE9 would arise from the coexistence of neurons expressing wild-type *PCDH19* and neurons non-expressing or expressing a mutant form of *PCDH19* [1, 69]. How would our GABAergic hypothesis fit into this model? The cell-interference model implies that *PCDH19* loss of function and related GABAergic defects involving all brain cells can be compensated for, possibly by homeostatic mechanisms and/or redundant proteins, for instance by other protocadherins that bind GABA_A_Rs [70]. By contrast, a mosaic pattern of *PCDH19* expression and related heterogeneously distributed GABAergic defects would have detrimental effects for neuronal connectivity. With this regard, pathogenic variant mosaicism has been reported in genes causing epilepsy-related neurodevelopmental disorders, including genes encoding for GABA_A_R subunits [71]. Mechanistically, it is tempting to speculate that a heterogeneous GABAergic tone between PCDH19-positive and PCDH19-negative cells might introduce, together with a scrambled PCDH19 adhesive code, a bias in cell-sorting patterns during brain development, and successively affect memory traces, which rely on the wiring of sparsely distributed neurons sharing the same excitatory level [72–74].

In conclusion, this study expands the knowledge of PCDH19’s role in the modulation of GABA_A_R-mediated transmission, and sheds light on novel mechanisms in the pathogenesis of EIEE9. The wide influence of PCDH19 on GABA_A_Rs is noteworthy. Altogether, the previous [10] and present findings demonstrate that PCDH19 is able to influence both components of GABAergic transmission, i.e., phasic and tonic inhibition. Moreover, PCDH19 demonstrated to act at different levels, regulating not only the amount of GABA_A_Rs that reaches the neuronal surface but also the gating of surface receptors. Our findings provide a key to interpret the complex EIEE9 clinical phenotype and to plan future specific therapeutic approaches.

## Electronic supplementary material

ESM 1 (PDF 4.54 mb)
