# Supplementary material for: The Epilepsy-Related Protein PCDH19 Regulates Tonic Inhibition, GABAAR Kinetics, and the Intrinsic Excitability of Hippocampal Neurons
Source: Mol Neurobiol. 2020 Sep 3;57(12):5336–51. doi: 10.1007/s12035-020-02099-7 (PMC7541378; doi:10.1007/s12035-020-02099-7)
Supplement: Supplementary file 1 — (PDF 4.54 mb) [file 12035_2020_2099_MOESM1_ESM.pdf]

Supplementary data

Figure S1

a **b**

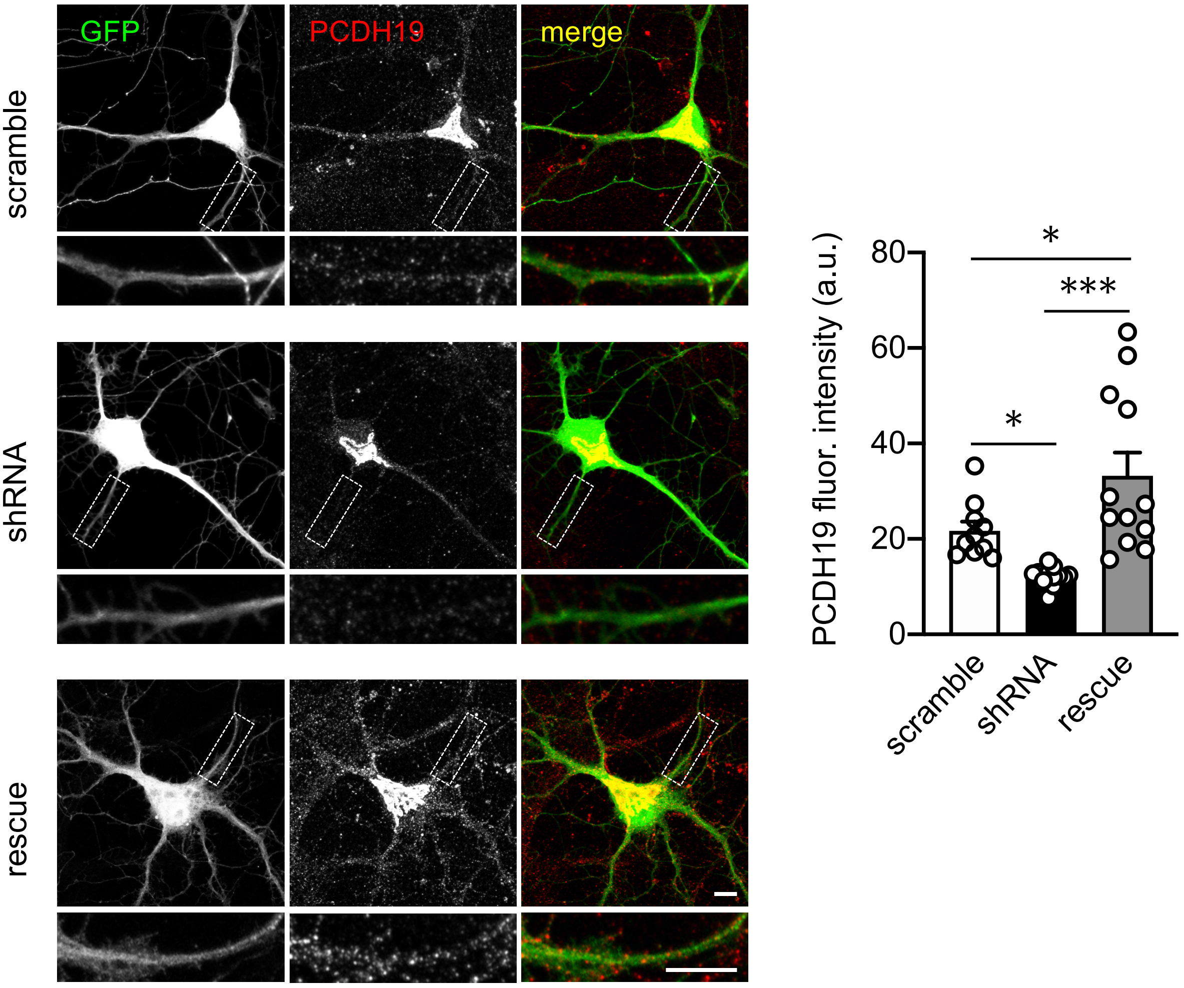

Legend to Figure S1

Fig. S1 Validation of PCDH19 shRNA and rescue strategy. **a** Representative confocal images of hippocampal neurons at DIV13 expressing control shRNA (scramble), PCDH19 shRNA (shRNA), and shRNA + PCDH19 (rescue). Transfected neurons were identified by GFP fluorescence and immunolabeled with anti-PCDH19 antibody. Insets show higher magnification of the dendrites framed by white rectangles. Scale bar, 10 μm. **b** Quantification of PCDH19 mean fluorescence intensity (in arbitrary units, a.u.) along neuronal dendrites. ShRNA-expressing neurons display a significant reduction of PCDH19 dendritic expression, while rescue neurons express on average more PCDH19 with respect of scramble neurons (scramble 21.71 ± 1.89, shRNA 12.16 ± 0.56, rescue 33.26 ± 4.84; one-way ANOVA, $F (2, 31) = 12$, $p = 0.0001$; Holm Sidak's *post hoc* test: scramble vs shRNA * $p = 0.042$, scramble vs rescue * $p = 0.031$; shRNA vs. rescue *** $p < 0.001$; N = 10-12 neurons per condition). Error bars are mean ± SEM

Figure S2

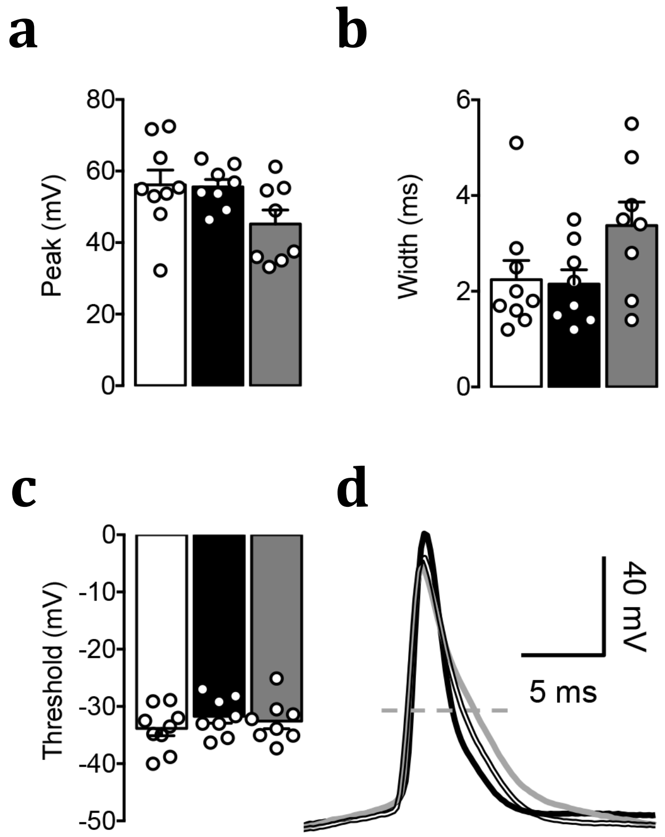
a
b
c
d
Peak (mV)
80
60
40
20
0
Width (ms)
6
4
2
0
Threshold (mV)
0
-10
-20
-30
-40
-50
40 mV
5 ms

Legend to Figure S2

Fig. S2 a – c Quantification of action potential (AP) peak (a), width (b) and threshold (c) in neurons expressing scramble, shRNA and rescue (AP peak, mV: scramble 56.13 ± 4.123, shRNA 55.56 ± 2.110, rescue 45.23 ± 3.902; one-way ANOVA, F (2, 22) = 2.919, p = 0.0751; AP width, ms: scramble 2.244 ± 0.3983, shRNA 2.150 ± 0.2994, rescue 3.375 ± 0.490; one-way ANOVA, F (2, 22) = 2.777, p = 0.084; AP threshold, mV: scramble -33.84 ± 1.275, shRNA -31.74 ± 1.192, rescue -32.56 ± 1.323; one-way ANOVA, F (2, 22) = 0.7192, p = 0.4982; N = 8-9 neurons per condition from 3-4 different cultures). Error bars are mean ± SEM. **d** Representative APs from scramble (white), shRNA (black) and rescue (gray) neurons showing that AP amplitude, width and voltage threshold remain unaltered following PCDH19-downregulation. Dotted gray line represents 0 mV

Table S1

a Firing frequency (Hz), basal condition (- BIC)

	Current (pA)							
	**10**	**20**	**30**	**40**	**50**	**60**	**70**	**80**
**scramble**								
**1**	0	0	0.5	0.75	2.75	3.75	n.d.	n.d
**2**	0	0	0	0	0.25	3	3.25	2.5
**3**	0	0	0.5	4	7.5	4.5	2	1.5
**4**	0	0	0.25	0.75	1	2.5	3.5	3.25
**5**	0	0	0	0	0	0	0	0.25
**6**	0	0	0	0	0	0.25	1	2
**7**	0	0	1	3.75	5	5.5	6.25	5
**8**	0	1.5	2	2	1.75	2.25	2.25	2.5
**9**	0	0	0	0	1.5	3	3.75	4.25
**10**	0	0	0	0	0.25	0.5	0.25	0.5
**11**	0	0	0.25	3.5	5	5.25	8.5	1.5
**12**	0	0	0.5	1.75	1.5	3	2.5	2.75
**13**	0	0	0	0	0.75	0.75	2.25	1
**14**	0	0	4.5	6	8.5	9	10.75	11
**15**	0	0	0	0.5	1.25	2.25	3	2.25
**16**	0	0	0	0.25	0.5	1.25	1.5	2.5
**shRNA**								
**1**	0	0	0.5	2.5	3.75	5.5	6.5	2.5
**2**	0	0	5.75	5	3.75	n.d.	n.d.	n.d.
**3**	0	0	2.75	3.75	5	4.5	4.5	4.25
**4**	0	0	5	6.25	7.25	7.5	5.5	2.75
**5**	0	0	2.5	4	4.25	5	5.5	5.25
**6**	0	0	0	0.25	1	3.25	3.75	5
**7**	0	0	1.25	4.75	5	5.5	5.5	5
**8**	0	1.5	1.25	2.75	3	3.5	4	4.5
**9**	0	0	0	0.25	0.5	2.5	10.5	12.75
**10**	0	0	2.5	4	3.75	10.25	n.d.	n.d.
**11**	0	0	2.75	5.75	6	6.5	6	7.25
**12**	0	0	2	1.75	1.75	2.25	1.5	2.75
**13**	0	0	0.25	0.5	1.25	1.25	1.5	2.75
**14**	0	0	0.5	1.25	2.5	4.75	9.75	12.75
**15**	0	0	5.75	13.25	15	12.5	2	1
**16**	0	0	1.5	2	3.25	2.75	4.5	6

rescue								
**1**	0	0	0	0	0.5	1.5	1	1
**2**	0	0	2.5	5	5	5.75	6.5	6.5
**3**	0	0	1	3.75	7.5	9.5	6.5	4
**4**	0	0	0	0.5	0.75	0.75	0.75	0.5
**5**	0	0	0	1.5	3.75	7.5	8.75	9.25
**6**	0	3	2	2.5	4	5	3.75	0.5
**7**	0	0	0	0.25	1	1	1.25	1
**8**	0	4	1.75	1.75	2	1.5	1.5	1.75
**9**	0	4	4.25	2.75	2.25	0.25	0.25	0.25
**10**	0	0	0.75	3.25	9	12.5	12	10.5
**11**	0	0	0	0	2.5	3.5	4	3.25
**12**	0	0	1.75	2	1.75	2.25	3.25	4.25
**13**	0	5	1.25	2.5	7.5	8	8	8
**14**	0	0	0	0	1	2	3.75	5
**15**	0	14	4.75	6.25	9	8.75	7.75	6.25
**16**	0	0	0	1.25	1.25	1	1.5	2
**17**	0	0	0	0	0.25	1	1.5	1.5

b Firing frequency (Hz), bicuculline (+ BIC)

	**Current (pA)**							
	**10**	**20**	**30**	**40**	**50**	**60**	**70**	**80**
**scramble**								
**1**	0	1	3.25	6	7.25	8.25	9	9
**2**	0	0.5	3.25	5.75	7.5	6.75	4	1.75
**3**	0.75	2	5	5	2.75	4	3.75	3.25
**4**	0	0.5	5	5.5	4.5	2.25	0.25	0.25
**5**	0	0	0	0	0.25	1.25	3.5	2.75
**6**	0	0	0.75	3.75	5.5	4.25	1.75	2
**shRNA**								
**1**	0	0.5	2	0.75	0.5	0.25	0.25	0.25
**2**	0	0	0	0.75	2.25	9.5	6.75	3.25
**3**	0	0	0	1	2.5	6	5	10.25
**4**	0	0	0	0.75	2	2.5	3	3.25
**5**	0	0	0	0	0.25	0.25	1.5	4.25
**6**	0	0	5	4.75	4.75	6.5	7.25	7.75
**7**	0.75	1.25	0.5	1.5	3.75	5.75	15.5	17.5
**8**	0	0	3.25	12.25	16.5	15	8.75	8.75
**9**	0	3	2.75	4.25	7	6.25	8	9
**10**	0	0	0.5	1.5	4	3.75	5.25	7.75

**rescue**								
**1**	0	0	0	0	0	0.75	1	1.25
**2**	0	0	0.25	2.25	4	4.25	5.5	5.75
**3**	1.5	7.75	11.5	3.5	1.5	1.25	1	0.75
**4**	0	0	0.5	3.5	5	4	4.25	7
**5**	0	0	3	3	8	6.25	7.5	10.75
**6**	0	0	0	0	0	0.25	1.25	1.25
**7**	0	0	4.75	11	10.25	3	2.25	1.25

Legend to Table S1

Table S1 Firing frequency data used for Figure 5, panels e and f. The firing frequency (Hz) at different current values (10 - 80 pA) is indicated for each neuron of scramble, shRNA, and rescue groups, under basal condition (**a**, top table, - BIC) or bicuculline bath-application (**b**, bottom table, + BIC). n.d., not detected
